# Probiotic supplementation and systemic inflammation in relapsing-remitting multiple sclerosis: A randomized, double-blind, placebo-controlled trial

**DOI:** 10.3389/fnins.2022.901846

**Published:** 2022-09-20

**Authors:** Mehran Rahimlou, Shima Nematollahi, Durdana Husain, Nasrin Banaei-Jahromi, Nastaran Majdinasab, Seyed Ahmad Hosseini

**Affiliations:** ^1^Nutrition and Metabolic Disease Research Center, Clinical Sciences Research Institute, Ahvaz Jundishapur University of Medical Sciences, Ahvaz, Iran; ^2^Department of Nutrition, Faculty of Medicine, Zanjan University of Medical Sciences, Zanjan, Iran; ^3^Department of Nutrition, School of Allied Medical Sciences, Ahvaz Jundishapur University of Medical Sciences, Ahvaz, Iran; ^4^Department of Neurology, School of Medicine, Ahvaz Jundishapur University of Medical Sciences, Ahvaz, Iran

**Keywords:** probiotic, multiple sclerosis, inflammation, clinical trial, gut microbiome

## Abstract

**Background:**

Multiple sclerosis (MS) is a complex inflammatory disease in which demyelination occurs in the central nervous system affecting approximately 2.5 million people worldwide. Intestinal microbiome changes play an important role in the etiology of chronic diseases.

**Objective:**

This study aimed to investigate the effect of probiotic supplementation on systemic inflammation in patients with MS.

**Methods:**

A 12-week double-blind clinical trial study was designed and seventy patients with MS were randomly divided into two groups receiving probiotics and placebo. Patients in the intervention group received two capsules containing multi-strain probiotics daily and patients in the control group received the same amount of placebo. Factors associated with systemic inflammation were assessed at the beginning and end of the study.

**Results:**

Sixty-five patients were included in the final analysis. There was no significant difference between the two groups in terms of baseline variables except for the duration of the disease (*P* > 0.05). At the end of the study, probiotic supplementation compared to the placebo caused a significant reduction in the serum levels of CRP (−0.93 ± 1.62 vs. 0.05 ± 1.74, *P* = 0.03), TNF-α (−2.09 ± 1.88 vs. 0.48 ± 2.53, *P* = 0.015) and IFN-γ (−13.18 ± 7.33 vs. −1.93 ± 5.99, *P* < 0.001). Also, we found a significant increase in the FOXP3 and TGF-β levels in the intervention group (*P* < 0.05).

**Conclusion:**

The results of our study showed that supplementation with probiotics can have beneficial effects on serum levels of some factors associated with systemic inflammation.

**Clinical trial registration:**

[http://www.irct.ir], identifier [IRCT20181210041 918N1].

## Introduction

Multiple sclerosis (MS) is a chronic disease characterized by inflammation and demyelination of the central nervous system (CNS) associated with variable degrees of axonal and neuronal damage and it’s most commonly diagnosed in people in their 20s, 30s and 40s although it can develop at any age (Tsang and Macdonell, 2011; Nourbakhsh and Mowry, 2019). The prevalence of this disease is higher among women, so that more than 60% of cases are women (Dörr et al., 2013). The predominant feature of this disease is the progressive demyelinating of to central nervous system neurons (CNS) following T cell mediated autoimmune processes (Tuller et al., 2011).

The results of previous studies have shown that various genetic and environmental factors are involved in the etiology of MS (Casaccia-Bonnefil et al., 2008). Various researchers have reported that MS is the result of an imbalance between inflammatory and anti-inflammatory conditions (Loma and Heyman, 2011). The permeability of the Blood-Brain Barrier (BBB) can be increased by pro-inflammatory cytokines, enabling neurodegeneration and demyelination of the CNS, while anti-inflammatory cytokines can suppress the release of pro-inflammatory cytokines (Hashemi et al., 2020). For these reasons, inflammation is one of the main components involved in the pathogenesis of MS and degeneration of brain axons and neurons (Morshedi et al., 2019). Among the various inflammatory factors, some proinflammatory cytokines, such as nterleukin-2 (IL-2), tumor necrosis factor- α (TNF-α) or interferon-g (IFN-g) which are secreted by T helper 1 (Th1) cells, and some anti-inflammatory cytokines such as Transforming growth factor beta 1 (TGF-β1) and interleukin 10 (IL-10) play an important role in the MS pathogenesis (Mirshafiey and Mohsenzadegan, 2009). In particular, interleukin (IL)-23–polarized T helper (Th)17 cells coexpressing T-bet and RAR-related orphan receptor gamma (RORγ) are considered pathogenic in both EAE (Codarri et al., 2011; Luchtman et al., 2014) and in MS (Luchtman et al., 2014). Interestingly, in MS subjects, reduced Th17 responses after hematopoietic stem cell transplantation was reported (Darlington et al., 2013). Th17 cells are characterized by the production of a large number of pro-inflammatory cytokines which include mainly IL-17A (also called IL-17), IL-17F, tumor necrosis factor-α (TNF-α), IL-21, IL-22, CCL20 and granulocyte monocyte-colony stimulating factor (GM-CSF) (Melnikov et al., 2020).

It has been reported that in these patients, we see an increase in the differentiation of naive CD4+ T into inflammatory cells and an increase in the ratio of cytotoxic T cells to immune-protective regulatory T (Treg) cells (Lowther and Hafler, 2012; Buc, 2013). Forkhead box P3 (FoxP3)+Treg cells play an essential role in immune system hemostasis and maintenance of self-tolerance (Cassani et al., 2012) and the results of some studies have shown that FoxP3 expression is impaired in these patients (Huan et al., 2005; Jafarzadeh et al., 2015).

In recent years, much attention has been paid to the association of intestinal microbiome with neurodegenerative diseases. Several studies have shown that microbiome integrity is impaired in patients with MS and the bacterial balance shifts to pathogenic bacteria (Cantarel et al., 2015; Chen et al., 2016). Some animal studies have also shown that the progression of experimental allergic encephalomyelitis (EAE), which is an animal model of MS, is more severe in Germ-free mice (Ochoa-Repáraz et al., 2009).

On the other hand, probiotic supplementation in some studies has significantly reduced the level of inflammatory factors and exerted inhibitory effects on inflammatory pathways (Steed et al., 2010; Kouchaki et al., 2017). Considering that inflammation is an integral part of the MS pathogenesis and reducing the level of inflammation can be effective in the treatment of this disease, this study aimed to investigate the effect of probiotic supplementation on systemic inflammation in patients with MS.

## Materials and methods

The method of this study was based on ethical standards from the Helsinki Declaration, approved by the ethics committee of Ahvaz Jundishapur University of Medical Sciences (IR.AJUMS.REC.1398.865) and registered on the Iranian Registry of Clinical Trials (IRCT) http://www.irct.ir: IRCT20181210041918N1). Informed consent was obtained from all patients.

### Study design and participants

The present study was a 12-week randomized, double-blind clinical trial which conducted between July 2019 and April 2020. The participants enrolling in this trial were patients referred to the MS association of Khuzestan, Iran, who were included in the study if they met the following criteria: age range between 18 and 50, clinical definite MS according to McDonald criteria and an Expanded Disability Status Scale (EDSS) score ≤ 4.5. Exclusion criteria included the presence of any concomitant disease such as rheumatoid arthritis, Inflammatory bowel disease (IBD), rheumatoid arthritis, systemic lupus, type 1 diabetes and other autoimmune diseases, use of anti-inflammatory drugs, omega-3 and probiotic supplements or other antioxidant and anti-inflammatory supplements, corticosteroid therapy, current smoker, disease duration of less than 1 year, malnourishment (BMI under 18.5) or morbid obesity (BMI > 35), impaired Th1/Th2 balance, (such as asthma, rheumatoid arthritis and type 1 diabetes mellitus), pregnancy and lactation. All patients were under the guidance of two neurologists and no restrictions were set on concomitant immunomodulatory treatment (i.e., Interferon beta-1a (IFN β-1a) (Avonex), glatiramer acetate, or natalizumab).

Finally, 70 patients with MS were randomly allocated in the intervention and control groups, so that 35 patients were divided into the intervention group and 35 patients into the control group. Randomization lists were computer-generated by a statistician and given to the interviewer. The randomization process took place in a way that researchers, neurologist, staff and patients were blinded.

Patients which participated in the study were randomly divided into two groups receiving probiotic supplement and placebo. Patients in the intervention group received 2 multi-strain probiotic capsules/day for 12 weeks, each containing minimum 2 billion live microor-ganisms (2 × 10^9^ CFU/capsule), equivalent to 10 billion live microorganisms per gram (1 × 10^10^ CFU/gram) of 14 strains (*Bacillus subtilis PXN 21, Bifidobacterium bifidum PXN 23, Bifidobacterium breve PXN 25, Bifidobacterium infantis PXN 27, Bifidobacterium longum PXN 30, Lactobacillus acidophilus PXN 35, Lactobacillus delbrueckii ssp. bulgaricus PXN 39, Lactobacillus caseiPXN 37, Lactobacillus plantarum PXN 47, Lactobacillusrhamnosus PXN 54, Lactobacillus helveticus PXN 45, Lactobacillus salivarius PXN 57, Lactococcus lactis ssp.lactis PXN 63, Streptococcus thermophilus PXN 66*), cellulose (bulking agent) and vegetable capsule (Hydroxy-propylmethyl Cellulose). Participants in the control group received the same amount of placebo for 12 weeks, and the placebo capsules contained microcellules, so that there was no difference in appearance or smell between probiotic and placebo capsules.

At the beginning of the study, after fully explaining the study protocol and receiving informed consent from patients, patients’ demographic information was recorded and then probiotic and placebo boxes were given to patients for 3 weeks. Subsequent probiotic and placebo boxes were presented to patients in the third, sixth and ninth weeks. So, in each visit (beginning of the study, third, sixth and ninth weeks), the patients received probiotics or placebo required for their consumption for three weeks (a package containing 42 capsules of probiotics or placebo) and this process was repeated again in the next visit at the end of the study. During the 12-week intervention period, patients were reminded of the use of probiotics and placebo by texting and calling. Patients who did not consumed more than 10% of the given capsules in each period were excluded from the study.

### Anthropometric measures, dietary intake and physical activity

To evaluate anthropometric variables, at the beginning and after week 12, patients’ height and weight were measured by a trained researcher. Patients ‘weight was measured using an Inbody device (Inbody BDM370, South Korea) without shoes, light clothing with the precision of 0.1 kg and patients’ height was measured using a Seca scale without shoes to the nearest 0.5 cm. The standard formula was also used to calculate body mass index (BMI).

To evaluate the dietary intake of the study participants, 3 days 24 h’ dietary recall on one holiday and two working days at the beginning and end of the study was recorded from all participants. The analysis of 24-h food recall questionnaires was done using Nutritionist IV (N4) (First Databank, Hearst Corp., San Bruno, CA, United States).

Physical activity was assessed by the metabolic equivalent of task (MET) questionnaire at the beginning and the end of the study.

### Serum biochemical measurement

To evaluate biochemical variables, at the beginning and end of the study, 10 cc of blood was taken from patients after 12 h of fasting. Blood samples were allowed to clot at room temperature (20-25°C) for 20 min in a vertical position. Then, the samples were centrifuged at 3000 rpm for 10 min and serum samples were frozen at −70°C, until biochemical marker measurement. ELISA kits (Diaclone Research, Besançon, France) were used to assess the concentration of Interferon gamma (IFNγ), Interleukin 17 (IL-17) and Interleukin 35 (IL-35). Also, for evaluation serum levels of TGF-β and FOXP3, we used from the Crystal Day Elisa kits (Shanghai Crystal Day Biotech, China). Measurements of plasma cytokines were done in double-antibody sandwich enzyme-linked immunosorbent assay (ELISA) method. Detection range for IFNγ was 12.5 pg/dl - 400 pg/dl, IL-17: 3.125 pg/dl - 1,000 pg/dl, IL-35: 3.25-2,000 pg/dl, TGF-β: 15.6-4,000 ng/ml and FOXP3 was 0.313 - 20 ng/ml. The ELISA intra-assay variation was evaluated by the CV of the duplicate measurements.

### Statistical analysis

All statistical analyses were performed with SPSS 19 software. Kolmogorov–Smirnov test was used to assess the normality of the data. Numerical data were presented as mean ± SD. Mann- Student’st-test was used to compare the continuous data and alternative non-parametric tests were used if the data not normally distributed. Categorical data were compared using the Chi-square test. Paired t-test or McNemar test was applied for intra-group comparison. Also, analysis of covariance was used to remove confounding variables. All of the analysis were adjusted for the age, sex, disease duration and calorie intake. *P* < 0.05 was considered significant.

## Results

**FIGURE 1:**
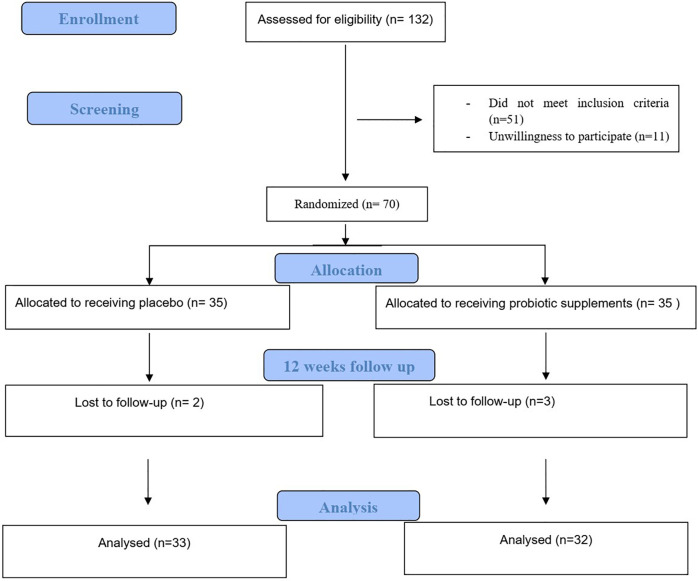
Study flow diagram.

The procedure of the trial is depicted in Figure 1 which summarizes the Consolidated Standards of Reporting Trials (CSRT). At the end of the 12 weeks of the intervention, out of 70 patients included in the study, three patients in the intervention group and two patients in the control group were excluded from the study for reasons such as travel, unwillingness to continue participating in the study and other reasons and finally 65 patients included in the final analysis (Schulz et al., 2010). Of 65 patients which included in final analysis, 33 participants (7 men and 26 women) in the probiotic group and 32 participants (10 men and 22 women) in the control group completed intervention period.

The demographic information of the study participant is reported in Table 1. There was no significant difference between the participants in the intervention and control groups in terms of baseline variables including age (*P* = 0.59), sex (*P* = 0.71), race (*P* = 0.47), weight (P = 0.65), height (*P* = 0.59), BMI (0.28) and MET (*P* = 0.34). In terms of EDSS score, the mean score of EDSS was 1.45 ± 0.9 in the intervention group and 1.39 ± 1.03 in the control group, but no significant difference was observed between the two groups (*P* = 0.74). In terms of disease duration, participants in the intervention group had higher disease duration in comparison to the control group (7.75 ± 3.99 y vs. 5.66 ± 2.53 y; *P* = 0.014).

**TABLE 1:** Baseline characteristics of the participants.

Variable		Total (*n* = 65)	Probiotic (*n* = 32)	Control (*n* = 33)	*P*-value
Age (years)		41.01 (10.45)	42.15 (11.98)	39.9 (8.76)	0.39
Height (cm)		165.56 (8.71)	164.96 (8.21)	166.13 (9.27)	0.59
Gender	Male	18 (27.7)	6 (18.75)	12 (36.36)	0.11
	Female	47 (72.3)	26 (81.25)	21 (63.64)	
Weight (kg)		68.7 (13.07)	69.45 (13.93)	67.96 (12.27)	0.65
WC (cm)		86.58 (11.86)	87.09 (11.29)	86.09 (12.54)	0.73
BMI (kg/m^2^)		25.01 (4.05)	25.48 (4.54)	24.55 (3.51)	0.36
MS duration (y)		6.69 (3.46)	7.75 (3.99)	5.66 (2.53)	0.014
EDSS Score		1.70 (0.72)	1.68 (0.71)	1.72 (0.74)	0.82
MET-h/day at study baseline		34.16 (4.84)	34.75 (4.95)	33.60 (4.75)	0.34

Data are presented as mean (SD) for quantitative and frequency (%) for qualitative variables. BMI: Body Mass Index; MS, Multiple sclerosis; EDSS, Expanded Disability Status Scale.

At the beginning of the study, the mean calorie intake by study participants in the intervention group was 1836.27 ± 464.71 kcal/day and in the control, group was 1,895 ± 398.07 kcal, which was not statistically significant (*P* = 0.58). Also, there wasn’t any significant difference between two groups in terms of calorie intake and macronutrients at the beginning and end of the study (*P* > 0.05). Moreover, there was no significant difference between the two groups in term of physical activity at the beginning and end of 12 weeks of intervention (*P* > 0.05).

### Effect of probiotic supplementation on serum levels of inflammatory markers

Effects of probiotics supplementation on the serum levels of inflammation related biomarkers are presented in the Table 2. As shown in Table 2, probiotic supplementation compared to the placebo caused a significant reduction in the serum levels of CRP (−0.93 ± 1.62 mg/dl vs. 0.05 ± 1.74 mg/dl, *P* = 0.03), TNF-α (−2.09 ± 1.88 pg/dl vs. 0.48 ± 2.53 pg/dl, *P* = 0.015) and IFN-γ (−13.18 ± 7.33 pg/dl vs. −1.93 ± 5.99 pg/dl, *P* < 0.001). In addition, the results of our study showed that there weren’t any significant differences between two groups in terms of IL-17 (*P* = 0.19) and IL-35 (*P* = 0.08) concentration. After adjusting the results for the confounding variables, no difference was observed in the significance of the results.

**TABLE 2:** Comparison of inflammatory biomarkers between groups at the baseline and end of the study^1^.

Variables^2^	Groups		*P*-value^3^	
	Intervention (*n* = 33)	Control (*n* = 32)		*P*- adjusted^4^
**IL-17 (pg/dl)**				
Before	25.43 ± 11.36	23.71 ± 9.65	0.24	
After	25.46 ± 14.49	25.04 ± 13.89	0.75	
Change	0.02 ± 1.19	1.32 ± 1.97	0.19	0.105
*P*-value^5^	0.32	0.18		
**IL-35 (pg/dl)**				
Before	10.29 ± 4.76	10.81 ± 6.12	0.16	
After	10.42 ± 5.16	11.16 ± 5.88	0.62	
Change	0.13 ± 0.65	0.35 ± 0.49	0.08	0.124
P-value^5^	0.376	0.182		
**CRP (mg/dl)**				
Before	3.62 ± 2.12	3.24 ± 1.28	0.43	
After	2.69 ± 1.78	3.29 ± 2.24	0.38	
Change	−0.93 ± 1.62	0.05 ± 1.74	0.03	0.034
P-value^5^	0.04	0.71		
**TNF-a (pg/dl)**				
Before	5.25 ± 3.28	4.68 ± 3.13	0.364	
After	3.16 ± 2.78	5.16 ± 3.62	0.089	
Change	−2.09 ± 1.88	0.48 ± 2.53	0.015	0.026
P-value^5^	0.021	0.367		
**IFN-γ** (**pg/dl**)				
BeforeAfterChange	33.17 ± 8.40 19.98 ± 2.57 −13.18 ± 7.33	30.76 ± 7.46 28.83 ± 7.14 −1.93 ± 5.99	0.17 < 0.001 < 0.001	< 0.001
P-value^5^	< 0.001	0.12		

1. CRP, C-reactive protein; IFN-γ, Interferon gamma, IL-17, Interleukin 17; IL-35, Interleukin 35; TNF-α, Tumor necrosis factor-α.

2. Data are presented as mean (SD) or geometric mean (SD).

3. Calculated using one-way ANOVA.

4. Calculated using ANCOVA, adjusted for the effect of age, sex, disease duration and calorie intake.

5. Calculated using paired sample *t*-test.

### Effect of probiotic supplementation on serum levels of TGF-β and FOXP3

The results of the effects of probiotic supplementation on serum levels of TGF-β and FOXP3 are shown in Table 3. The results of our study showed that probiotic supplementation caused a significant increase in the serum concentration of TGF-β1 (0.53 ± 0.67 ng/ml vs. −0.07 ± 0.58 ng/ml) compared to the placebo. Also, it has been reported that participants in the intervention group had significantly higher levels of FOXP3 (0.25 ± 0.41 ng/ml vs. −0.02 ± 0.53 ng/ml, *P* = 0.014).

**TABLE 3:** Comparison of TGF-β and FOXP3 levels between groups at the baseline and end of the study^1^.

Variables^2^	Groups		*P*-value^3^	
	Intervention (*n* = 33)	Control (*n* = 32)		*P*- adjusted^4^
**TGF-β** (**ng/ml**)				
Before	1.45 ± 0.52	1.83 ± 0.87	0.135	
After	1.98 ± 0.74	1.76 ± 0.65	0.55	
Change	0.53 ± 0.67	-0.07 ± 0.58	0.023	0.036
P-value^5^	0.03	0.16		
**FOXP3** (**ng/ml**)				
Before	1.42 ± 0.92	1.54 ± 0.73	0.182	
After	1.67 ± 0.91	1.52 ± 0.88	0.309	
Change	0.25 ± 0.41	-0.02 ± 0.53	0.014	0.026
P-value^5^	0.02	0.8		

1. FOXP3, forkhead box P3; TGF-β, Transforming growth factor beta.

2. Data are presented as mean (SD) or geometric mean (SD).

3. Calculated using one-way ANOVA.

4. Calculated using ANCOVA, adjusted for the effect of age, sex, disease duration and calorie intake.

5. Calculated using paired sample *t*-test.

## Discussion

MS is one of the most important inflammatory diseases and inflammation plays an important role in the pathogenesis of this disease. Numerous studies have shown that the concentration of inflammatory factors increases in patients with MS compared to healthy individuals. Nazeri et al in a case control study showed that the serum levels of high-sensitivity C-reactive protein (hs-CRP) was higher in patients with MS specially patients with cerebellar and brain stem symptoms (Nazeri et al., 2021). Also, Farrokhi M and Polachini in other studies found a significant increase in the serum levels of inflammatory markers, Like CRP in MS patients compared to the control group (Polachini et al., 2014; Farrokhi et al., 2017). However, Yoon et al. reported that CRP may be a useful marker for the detection of inflammation-mediated MS relapses (Yoon et al., 2013).

It has been reported that the destruction of the intestinal microbiome is one of the main causes of chronic inflammation and can accelerate the progression of MS (Mielcarz and Kasper, 2015; Jangi et al., 2016). One of the hypotheses that has been suggested in connection with the high prevalence of MS in most countries is the excessive use of antibiotics and especially the destruction of the intestinal microbiome following inappropriate changes in dietary patterns (Kirby and Ochoa-Repáraz, 2018). In fact, the results of various studies have shown that intestinal microbiome dysbiosis has been associated with the development of various immune-related diseases such as MS, rheumatoid arthritis, type 1 diabetes, and inflammatory bowel disease (Berer et al., 2011; Miyake et al., 2015; Jangi et al., 2016).

The present clinical trial demonstrated that the supplementation of multi strain probiotics resulted in a significant reduction in serum concentration of some inflammatory biomarkers including CRP, TNF-α and IFN-γ. However, there wasn’t any significant differences between two groups in term of IL-17. Our finding was similar to that reported in some previous studies. Most studies that have evaluated the effect of bacterial strains on the severity of MS attacks and inflammatory biomarkers have been studies on experimental model of MS (EAE).

Salehipour et al. (2017) in an animal study evaluated the effect of administering several bacterial strains, especially *plantarum A7* on experimental model of MS, and the results showed that mice receiving probiotics had a significant improvement in the level of anti-inflammatory factors, including TGF-β1 and FOXP3 and significant reduction in the serum levels of IL-17 and IFN-γ. Also, Secher et al. (2017) have shown that probiotic administration in mice model with EAE led to a significant reduction in the serum levels of IL-17, IFN-γ, and TNF-α. They suggested that the anti-inflammatory effects of probiotics could be exerted by reducing the number of total numbers of CD4^+^ and MOG-specific CD4^+^ T^–^ cells in the spinal cord (SC) regulatory T cells and increasing lethal T cells. In another animal study, Kwon et al. (2013) showed that administration of five bacterial strains in mice with EAE slowed the progression of disease and decreased the expression of inflammatory factors. Also, Lavasani et al. (2010) showed that administration of multi strain probiotics in mice with EAE was more effective in reducing inflammatory factors than single bacterial strain. Tamtaji et al. (2017) in a human study showed that probiotic supplementation in 20 patients with MS down-regulated the expression of interleukin-8 and TNF-α genes.

Various human and animal studies have shown that probiotics exert anti-inflammatory and immune-boosting effects through a variety of mechanisms, such as maintaining mucosal barrier integrity, improving mucus secretion, decrease in the number of lipopolysaccharides (LPS) and some other mechanisms (Forsyth et al., 2009; Bull-Otterson et al., 2013; Hosseinifard et al., 2019). LPS play an important role in the exacerbation of MS by binding to the toll-like receptors (TLR2, 4) on endothelial cells (ECs), DCs (dendritic cells) and macrophage cells (MQs). Stimulation of TLR2 and TLR4 by LPS increases the production and secretion of inflammatory cytokines (Erridge et al., 2008; Tankou et al., 2018a). In fact, probiotic supplementation has been shown to significantly increase the differentiation of native T cells to the Th2 which improve production and secretion of anti-inflammatory cytokines such as IL-10 and IL-4 (Lavasani et al., 2010; Kwon et al., 2013). It has been reported that Lactobacillus species oral administration in mice with EAE led to IL- 10-dependent activation of Tregs in the CNS followed by reduction of IFN-γ, TNF-α, and IL-17. and they concluded that improving the gut microbial profile could reduce chronic inflammation (Banati et al., 2013).

Some studies have reported that administration of probiotics, especially *Lactobacillus strains*, induces anti-inflammatory effects by reducing the number of intermediate monocytes (Tankou et al., 2018b). Also, some metabolites produced by probiotics, such as butyrate, induce anti-inflammatory effects by inhibition of the NF-kB pathway as well as inhibiting the secretion of lipopolysaccharide-induced TNF-α and IL-6 (Segain et al., 2000). Zhang et al. showed that administration of lactobacillus rhamnosus in Caco-2 cells causes a significant reduction in the TNF-α induced IL-8 secretion (Zhang et al., 2005). Also, Shimazu et al. in a cell study reported that porcine intestinal epithelial cells treatment with *lactobacillus jensenii* led to a significant anti-inflammatory effects by down-regulating the NF-kB and mitogen-activated protein kinase (MAPK) pathways (Wu et al., 2016).

We found that probiotic supplementation for 12 weeks led to a significant increase in the serum levels of FOXP3 and TGF-β1. As mentioned, MS results from the failure of the body’s regulatory mechanisms, such as regulatory T cells against the spread of pathogenic T cells directed at myelin determinants. Among the regulatory cells that play a protective role against the progression of MS are CD4^+^CD25^+^ regulatory T cells. CD4^+^CD25^+^ cells have a unique function and support both central and peripheral tolerance in the body (Hashemi et al., 2020). These cells exert central tolerance in the thymus and also induce peripheral Tregs (Walker et al., 2003). On the other hand, CD4^+^CD25^+^ cells play an important role in inhibiting effector T-cell proliferation as well as reducing the production and secretion of inflammatory cytokine in a cytokine-independent way requiring cell-to-cell contact. The FOXP3 transcription factor is one of the most important and sensitive indicators for evaluation of CD4^+^CD25^+^ regulatory T cells. It has been reported that in patients with MS, the expression and function of FOXP3 is significantly impaired (Huan et al., 2005). On the other hand, the results of some studies have shown that any mutation in FOXP3-related genes disrupts the development of regulatory T cells and increases the risk of some autoimmune diseases such as MS, some inflammatory and allergic diseases (Hori and Sakaguchi, 2004).

Smelt et al. in an animal study were reported that oral administration of *L. plantarum WCFS1, L. salivarius UCC118, and L. lactis MG* in healthy mice led to a significant increase in the FoxP3 T-cell responses in the small intestine and simultaneously inducing CD4 and CD8 T cell activation in the large intestine (Smelt et al., 2013). Also, Lavasani et al. in an animal study evaluated the effects of five bacterial strains in mice with EAE and showed that probiotic oral administration induced CD4^+^CD25^+^Foxp3^+^ regulatory T cells (Tregs) in mesenteric lymph nodes and also improved TGF-β1 levels (Lavasani et al., 2010).

TGF-β1 is one of the most important regulatory cytokines that has a variety of functions in the immune system as an anti-inflammatory agent and influence the differentiation and function of T cells. One of the most important roles of insulin is to promote immune self-tolerance by regulation of lymphocyte proliferation, differentiation, and survival. Among T cells, CD4^+^CD25^+^FOXP3^+^ T regs contain the main source of TGF-β1 that suppresses immune responses in inflammatory sites (Mirshafiey and Mohsenzadegan, 2009; Lee et al., 2017). In line with our findings, some other studies showed the positive effects of probiotic administration on TGF-β1 concentration (Foye et al., 2012; Morshedi et al., 2019; Takahashi et al., 2019).

### Strengths and limitations

As mentioned, in recent years, limited studies have evaluated the effect of probiotics on patients with MS, and our study was one of the most recent trials in this field. One of the main strengths of our study was the use of multi-strain probiotic supplements instead of a single specific bacterial strain. Various studies have shown that the use of products containing several bacterial strains is more effective in improving inflammation and strengthening the immune system than a single bacterial strain (Whorwell et al., 2006; Kobyliak et al., 2018; Ouwehand et al., 2018). Also, the duration of intervention in the present study was long and it seems that the duration of intervention of 12 weeks causes appropriate changes in the intestinal microbial profile. On the other hand, unlike some previous studies, the participants in this study included both men and women, and therefore the results of the study can be generalized to both sexes.

However, the present study had some limitations that should be considered in interpreting the results. One of the main limitations of our study was the lack of evaluation of changes in intestinal microbial profiles in fecal samples. Also, assessing the gene expression of some inflammatory factors, especially TGF-β1 and FOXP3, could increase the accuracy of the results.

## Conclusion

In conclusion, the results of our study revealed that the modification of the gut microbiota to a more favorable composition may contribute to improved systemic inflammation in patients with MS. Despite the positive results in this study, more studies are needed to prove the findings of this study. Identification of the mechanisms involved in these beneficial effects can also be considered in the design of future studies.

## Data availability statement

The original contributions presented in the study are included in the article, further inquiries can be directed to the corresponding author/s.

## Ethics statement

The studies involving human participants were reviewed and approved by Ahvaz Jundishapur University of Medical Sciences. The patients/participants provided their written informed consent to participate in this study.

## Author contributions

MR and SH designed the research. MR, SN, and NB-J conducted the research. NM analyzed the data. MR and DH wrote the article. SH had primary responsibility for the final content. All authors read and approved the final manuscript.

## Abbreviations

BBB: blood-brain barrier
BMI: body mass index
CRP: C-Reactive Protein
CSRT: Consolidated Standards of Reporting Trials
DCs: dendritic cells
IFN-g: interferon-g
IL-10: interleukin 10
MQs: macrophage cells
FOXP3: Forkhead box P3
MAPK: mitogen-activated protein kinase
NF-kB: nuclear factor kappa-light-chain-enhancer of activated B cells
Treg: regulatory T cells
EAE: experimental allergic encephalomyelitis
ECs: endothelial cells
IRCT: Iranian Registry of Clinical Trials
EDSS: Expanded Disability Status Scale
IBD: Inflammatory bowel disease
MS: Multiple sclerosis
MET: metabolic equivalent of task
IL-17: Interleukin 17
IL-35: Interleukin 35
TLR: toll-like receptors
TGF-β: Transforming growth factor beta
TNF-a: tumor necrosis factor-a.
