# Corrigendum: Probiotic supplementation and systemic inflammation in relapsing-remitting multiple sclerosis: A randomized, double-blind, placebo-controlled trial

**DOI:** 10.3389/fnins.2022.1085572

**Published:** 2022-11-15

**Authors:** Mehran Rahimlou, Shima Nematollahi, Durdana Husain, Nasrin Banaei-Jahromi, Nastaran Majdinasab, Seyed Ahmad Hosseini

**Affiliations:** ^1^Nutrition and Metabolic Disease Research Center, Clinical Sciences Research Institute, Ahvaz Jundishapur University of Medical Sciences, Ahvaz, Iran; ^2^Department of Nutrition, Faculty of Medicine, Zanjan University of Medical Sciences, Zanjan, Iran; ^3^Department of Nutrition, School of Allied Medical Sciences, Ahvaz Jundishapur University of Medical Sciences, Ahvaz, Iran; ^4^Department of Neurology, School of Medicine, Ahvaz Jundishapur University of Medical Sciences, Ahvaz, Iran

**Keywords:** probiotic, multiple sclerosis, inflammation, clinical trial, gut microbiome

In the published article, there was an error in affiliation 1 as published. Instead of “1 Department of Nutrition, School of Allied Medical Sciences, Ahvaz Jundishapur University of Medical Sciences, Ahvaz, Iran,” it should be “1 Nutrition and Metabolic Disease Research Center, Clinical Sciences Research Institute, Ahvaz Jundishapur University of Medical Sciences, Ahvaz, Iran.”

In addition, there was an error in the affiliations for Nasrin Banaei-Jahromi. Instead of “1 Department of Nutrition, School of Allied Medical Sciences, Ahvaz Jundishapur University of Medical Sciences, Ahvaz, Iran,” it should be “3 Department of Nutrition, School of Allied Medical Sciences, Ahvaz Jundishapur University of Medical Sciences, Ahvaz, Iran.”

The authors apologize for this error and state that this does not change the scientific conclusions of the article in any way. The original article has been updated.

## Publisher's note

All claims expressed in this article are solely those of the authors and do not necessarily represent those of their affiliated organizations, or those of the publisher, the editors and the reviewers. Any product that may be evaluated in this article, or claim that may be made by its manufacturer, is not guaranteed or endorsed by the publisher.

